# Effect of oxygen tension and antioxidants on the developmental competence of buffalo oocytes cultured *in vitro*

**DOI:** 10.14202/vetworld.2021.78-84

**Published:** 2021-01-11

**Authors:** Amro M. El-Sanea, Ahmed Sabry S. Abdoon, Omaima M. Kandil, Nahed E. El-Toukhy, Amal M. Abo El-maaty, Hodallah H. Ahmed

**Affiliations:** 1 Department of Animal Reproduction and Artificial Insemination, Veterinary Research Division, National Research Centre, Tahrir St., Dokki 12622, Cairo, Egypt; 2 Department of Animal Physiology, Faculty of Veterinary Medicine, Cairo University, Giza Square 12211, Cairo, Egypt

**Keywords:** antioxidants, buffalo oocytes, developmental competence, *in vitro* embryo production, O_2_ tension

## Abstract

**Aim:**

Oxidative stress (OS) is one of the major disruptors of oocyte developmental competence, which appears due to the imbalance between the production and neutralization of reactive oxygen species (ROS).

**Materials and Methods:**

In Experiment 1, buffalo oocytes were *in vitro* matured, fertilized, and cultured at 38.5°C under 5% CO_2_ + 20% O_2_ in standard CO_2_ incubator (OS) or under 5% O_2_ + 5% CO_2_ + 90% N_2_ (Multi-gas incubator, low O_2_). In Experiment 2, buffalo cumulus oocytes complexes (COCs) were matured in Basic maturation medium (BMM) composed of TCM199+ 10% FCS+ 10 µg/ml FSH+ 50 µg/ml gentamicin (control group) or in BMM supplemented with 50 μM ascorbic acid (ascorbic acid group) or 3.0 mM glutathione (glutathione group) or 10^-5^ M melatonin (melatonin group) and cultured at 38.5°C under 20% O_2_ for 24 h. Matured buffalo oocytes in control, ascorbic acid, or melatonin groups were fertilized and zygotes were cultured for 8 days under the same conditions.

**Results:**

In both experiments, maturation, cleavage, and blastocyst rates were recorded. Results showed that culture of buffalo oocytes under low O_2_ (5% O_2_) significantly increased maturation, cleavage, and blastocyst rates (p<0.05). Meanwhile, under 20% O_2_, addition of 10^-5^ M melatonin or 50 μM ascorbic acid to *in vitro* maturation (IVM) medium significantly improved cumulus cell expansion, nuclear maturation rates of buffalo oocytes (p<0.05), and increased cleavage and blastocyst rates (p<0.05).

**Conclusion:**

About 5% O_2_ is the optimum condition for *in vitro* production of buffalo embryos, and addition of 10^-5^ M melatonin to IVM medium for oocytes cultured under 20% O_2_ could alleviate the adverse effect of high oxygen tension and increased embryo yield.

## Introduction

The application of assisted reproductive technologies such as *in vitro* embryo production (IVEP) and embryo transfer is slower in buffalo than in cattle. Oocytes matured *in vitro* showed lower quality and reprogramming competence compared to oocytes matured *in vivo* [1]. Oxidative stress (OS) during IVEP is one of the major disruptors of oocyte developmental competence; it appears due to the imbalance between the production and neutralization of reactive oxygen species (ROS) [2]. OS induces granulosa cell apoptosis and reduces the transfer of nutrients and growth factors to oocytes leading to apoptosis [3]. Moreover, buffalo oocytes possess higher lipid content as compared with many other species and they are very sensitive to OS, which leads to deterioration in oocyte quality and affect their developmental potentials [4].

OS of 20% O_2_ during IVEP was associated with higher H_2_O_2_ formation within the bovine embryos [5] and a reduction in the number of inner cell mass within the bovine blastocyst compared with 5% O_2_ tension [6]. Furthermore, 5% O_2_ tension during *in vitro* culture of embryos produced a higher blastocyst rate in buffalo [7] and cattle [8-10]. There is a lack of information on the effect of OS on maturation, fertilization, and blastocyst rates of *in vitro* produced buffalo embryos. Furthermore, a variety of antioxidants has been used to decrease ROS during the IVEP [11,12]; however, which antioxidant is the most suitable to support the development of buffalo embryos is still undefined. Melatonin ameliorates oocyte OS and improves subsequent *in vitro* development and decreased apoptosis levels, recovered the integrity of mitochondria, amend the spindle assembly and chromosome alignment in oocytes, and enhances embryo development in bovine [1,13], pigs [14,15], mice [16], and human [17]. In addition, antioxidant glutathione (GSH) plays an important role in the ­antioxidant system of cells [18]. The concentrations of GSH in matured oocytes were significantly higher than that in the immature oocytes and play an important role in successful fertilization [19]. Furthermore, *in vitro* maturation (IVM) of bovine oocytes in the presence of Vitamin C significantly increases the blastocyst rate [20]. Better understanding of the direct effect of manipulation of oxygen tension and addition of antioxidants to maturation medium could improve the developmental competence of buffalo oocytes.

Therefore, the present study was undertaken to investigate: (1) The effect of *in vitro* culture of buffalo oocytes and embryos under OS condition of 20% O_2_ or that of 5% O_2_ tension on the developmental competence of buffalo oocytes and (2) the effect of addition of antioxidants (50 μM ascorbic acid, 3.0 mM GSH, or 10^-5^ M melatonin) to IVM medium of buffalo oocytes cultured under 20% oxygen tension on cumulus cell expansion, nuclear maturation rates, and their ability develop to blastocyst stage.

## Materials and Methods

### Ethical approval

All animal studies in the present work were conducted in accordance with the requirement of the Institutional Animal Care Committee and were reviewed and approved by the Animal Ethics Committee of the National Research Centre of Egypt (NRC, ID: 12/1/7).

### Experimental procedures

All chemicals used in this study were purchased from Sigma-Aldrich (St. Louis, MO, USA), unless otherwise mentioned**.**

#### Experiment 1: Effect of OS (20% O_2_) on IVM and development rates of buffalo oocytes

Buffalo ovaries were collected and transported to the laboratory in thermos containing warm (32-35°C) saline solution. Cumulus oocytes complexes (COCs) were retrieved and classified into four categories based on number of cumulus cell layers and homogenous of the cytoplasm [21], and Grade 1 and 2 COCs were used for IVM. After selection, COCs were washed at least 3 times in phosphate-buffered saline supplemented with 4 mg/mL bovine serum albumin (BSA) + 50 μg/mL gentamicin, then washed at least 2 times in IVM basic medium (BMM), which consists of TCM-199 medium supplemented with 10% fetal calf serum + 10 μg/mL follicle-stimulating hormone + 50 IU equine chorionic gonadotropin (Merck, Germany) + 50 μg/mL gentamicin. Twenty-five to 50 COCs were cultured in 500 μL of BMM in 4-well culture plate (Nunc, Denmark). COCs were IVM at 38.5°C for 24 h in a standard CO_2_ incubator (Contherm, New Zealand) under 5% CO_2_ and 20% O_2_ (OS group) or in Multi-gas incubator (Binder, Germany) under 5% CO_2_, 5% O_2_, and 90% N_2_ (control group) in humidified air. This experiment was conducted at least 3 times.

**Figure-1:**
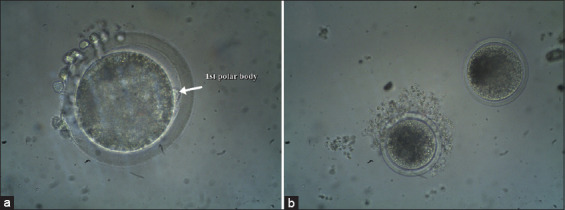
Photomicrograph showing *in vitro* matured buffalo oocytes at MII stage and extruding the 1^st^ polar body (white arrow, a, 200×) and non-matured oocytes without a polar body (b, 100×).

For the assessment of nuclear maturation, cumulus cells were removed by gentle pipetting and oocytes were examined under an inverted microscope (Axio Vert, Zeiss, Germany). Oocytes showing the 1^st^ polar body were considered as matured at metaphase II (M II) (Figure-1a) and oocytes without polar body were considered as non-matured oocytes (Figure-1b).

After IVM, matured oocytes were subjected to *in vitro* fertilization (IVF). Frozen buffalo semen was purchased from Animal Reproduction Research Institute; the same batch of the same bull with proven fertility was used throughout the study. One 0.25 mL straw of frozen semen was thawed in a water bath at 37°C for 30 s. Spermatozoa were washed twice by centrifugation at 180 g for 5 min in Sperm Tyrode’s albumin lactate pyruvate (Sp-TALP) medium supplemented with 4 mg/mL BSA + 50 μg/mL gentamicin. After washing, the sperm pellet was suspended in 200 μL Fert-TALP medium and the sperm number was counted using a hemocytometer. The final sperm concentration was adjusted to 2×10^6^/mL using Fert-TAPL medium supplemented 10 μg/mL heparin, 5 mg/mL BSA (fatty acid free) + 2.5 mM caffeine sodium benzoate + 50 μg/mL gentamicin. The sperm suspension (300 μL) was placed into a 4-well culture plate and covered with warm 200 μL mineral oil. Maturated buffalo oocytes were washed 3 times in Fert-TALP medium, and then, 15-20 oocytes were transferred into the sperm suspension droplet and cultured either under 5% CO_2_ and 20% O_2_ in standard CO_2_ incubator (OS group) or 5% CO_2_, 5% O_2_, and 90% N_2_ (Multi-gas incubator, control group) at 38.5°C for 18-20 h in humidified air.

**Figure-2:**
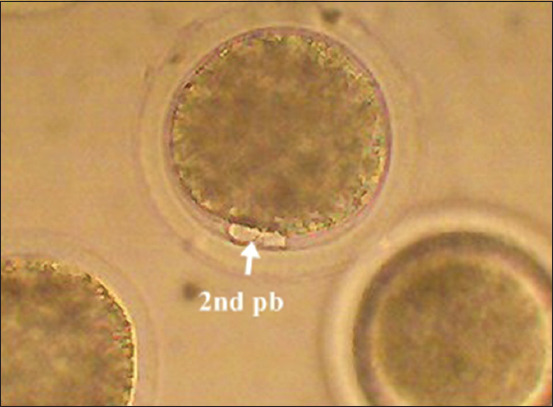
Fertilized buffalo oocytes showing the second polar body (200×).

The fertilization rate was checked by counting the number of oocytes extruding the second polar body (Figure-2). Then, the presumptive zygotes were washed twice and cultured in modified synthetic oviduct fluid (mSOF) medium supplemented with 5 mg/mL BSA + 50 μg/mL gentamicin for 8 days. Zygotes were culture under the same incubation conditions during IVM and IVF (5% or 20% O_2_ tension). Cleavage and embryo development rates were checked on days 2, 5, 7, and 8 using an inverted microscope.

#### Experiment 2: Effect of addition of 50 μM ascorbic acid, 3.0 mM GSH, or 10^-5^ M melatonin in IVM medium on maturation rate and development of buffalo oocytes

In six replicates, buffalo COCs were collected, evaluated and IVM in 500 μL BMM as in Experiment 1. Buffalo COCs were divided into four groups: (1) Control group, COCs were IVM in BMM; (2) ascorbic acid group, in which buffalo COCs were IVM in BMM supplemented with 50 μM ascorbic acid; (3) glutathione group, in which buffalo COCs were IVM in BMM supplemented with 3.0 mM GSH; and (4) melatonin group, in which buffalo COCs were IVM in BMM supplemented with 10^-5^ M melatonin. In all groups, buffalo COCs were incubated at 38.5°C for 24 h under 5% CO_2_ and 20% O_2_ (OS, in standard CO_2_ incubator) in humidified air.

#### Evaluation of oocytes cytoplasmic and nuclear maturation rates

**Figure-3:**
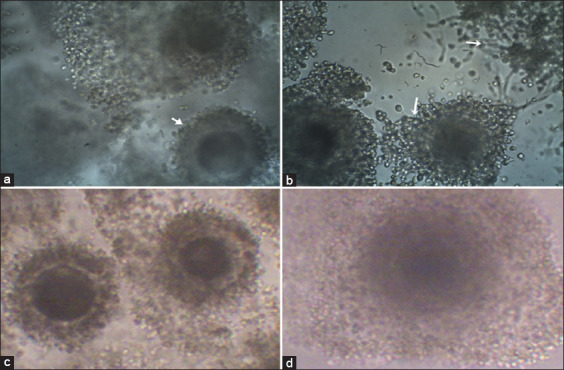
Photomicrograph showing the grade of cumulus cell expansion of *in vitro* maturation (IVM) buffalo oocytes; (a) Grade 0 showed no cumulus cell expansion (arrow); (b) Grade 1 with expansion of the outer layer of cumulus cells (arrow); (c) Grade 2: IVM buffalo oocytes with moderate cumulus cells expansion; and (d) Grade 3: IVM buffalo oocytes with full expansion of cumulus cells (100×).

After IVM, cytoplasmic maturation was checked in all groups based on the degree of cumulus cell expansion. Cumulus oocyte complexes were classified according to their degree of cumulus cell expansion into four grades. Grade 0 (G0), COCs with no cumulus cell expansion. Grade 1 (G1), COCs with slight expansion of the outer layer of cumulus cells, Grade 2 (G2), COCs with moderate expansion of cumulus cells, and Grade 3 (G3), COCs with full expansion of cumulus cells (Figure-3a-d). Assessment of nuclear maturation was performed as in Experiment 1. After evaluation of IVM rate, GSH group was excluded out from *in vitro* fertilization and *in vitro* culture due to the low maturation rate compared with the control group.

In control, ascorbic acid, and melatonin groups, IVM matured oocytes were subjected to IVF as in Experiment 1, and fertilized oocytes were transferred into the sperm suspension droplet (300 μL Fert-TALP medium) and incubated for 18-20 h at 38.5°C under 5% CO_2_ and 20% O_2_ (OS) in humidified air.

After IVF, oocytes were washed twice in mSOF medium supplemented with 5 mg/mL BSA + 50 μg/mL gentamicin for 8 das. Cleavage and embryo development rates were checked on days 2, 5, 7, and 8 using an inverted microscope.

### Statistical analysis

Data were expressed as the Mean ± SEM. Statistical analysis was performed using Student’s “t” test and analysis of variance (ANOVA) with the aid of SPSS 20.0 statistical software (SPSS Inc., Chicago, IL, USA). Duncan’s multiple range tests were used to differentiate between significant means at p<0.05.

## Results

### Experiment 1: Effect of OS condition on the developmental competence of buffalo oocytes

**Figure-4:**
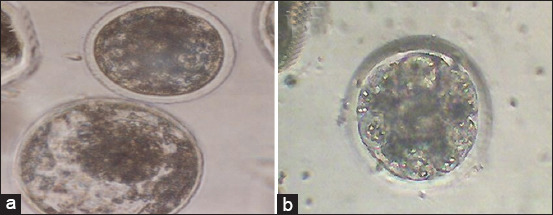
*In vitro* produced buffalo blastocyst produced under 5% O_2_ and 5% CO_2_ + 90% N_2_ (a, 200×) and morula stage derived after *in vitro* maturation, fertilization, and culture of buffalo oocytes under 20% oxygen and 5% CO_2_ (b, 200×).

The effect of OS (20% vs. 5% oxygen) on maturation rate and development of buffalo oocytes is illustrated in Table-1. Data revealed that IVM under 20% oxygen tension significantly (p<0.05) decreased maturation rate of buffalo oocytes. After IVF, cleavage rate was higher (p<0.05) for buffalo oocytes cultured under 5% CO_2_ + 5% O_2_ and 90% N_2_ than that cultured under 5% CO_2_ + 20% O_2_ (OS). Moreover, the percentage of 8-16 cell stage and morula stages did not vary for buffalo oocytes cultured under 5% or 20% O_2_ (Figure-4b). Meanwhile, the percentage of 2-4 cell stage was lower (p<0.05) and blastocyst rate (Figure-4a) was higher (p<0.05) for oocytes cultured under 5% CO_2_ + 5% O_2_ and 90% N_2_ than that cultured under OS conditions of 5% CO_2_ + 20% O_2_. These results indicated that 5% CO_2_+ 5% O_2_ support the development of *in vitro* produced buffalo embryos to the blastocyst stage better than 20% O_2_ conditions.

**Table-1:** Effect of oxygen tension during *in vitro* culture on maturation and developmental rates of buffalo oocytes (Mean±SEM).

Group	Number of oocytes	Maturation rate (%)	No. of fertilized oocytes	Embryo development rate %				

				Fertilization rate (%)	Embryo development rate (%)			

					2-4 cell	8-16 cell	Morula	Blastocyst
5% oxygen	274	85.0±1.3 (233)	212	82.5±2.0 (175)^a^	26.2±1.6^b^	21.1±1.2	28.3±1.1	24.4±1.3^a^
20% oxygen	246	72.4±1.0 (178)	178	73.6±1.5 (131)^b^	37.83±1.4^a^	21.2±1.2	28.4±1.8	13.6±1.6^b^

^a,b^Superscripts within the same column differ significantly at p<0.05.

### Experiment 2: Effect of addition of 50 μM ascorbic acid, 3.0 mM GSH, or 10^-5^ M melatonin in IVM medium on maturation and development of buffalo oocytes

The effect of addition of 50 μM ascorbic acid, 3.0 mM GSH, or 10^-5^ M melatonin to IVM medium on cumulus cell expansion and nuclear maturation rate of IVM buffalo oocytes is demonstrated in Table-2. Results showed that the addition of 10^-5^ M melatonin to maturation medium of buffalo oocytes significantly (p<0.05) increased the percentage of oocytes with Grade 3 cumulus cell expansion (G3, full cumulus cell expansion). Melatonin also increased oocyte nuclear maturation (MII stage) compared to control oocytes or those that were matured in the presence of 50 μM ascorbic acid or 3.0 mM GSH (p<0.05). Addition of 3.0 mM GSH to IVM medium of buffalo oocytes significantly (p<0.05) decreased the percentage of G3 cumulus cell expansion and nuclear maturation rate compared with the control group.

**Table-2:** Effect of antioxidant supplementation into *in vitro* maturation medium on cumulus cells expansion and nuclear maturation of buffalo oocytes.

Group	No. of oocytes	Cumulus cell expansion (%)				Nuclear maturation (%)	
	
		G0	G1	G2	G3	Mature	Non-mature
Control (BMM)	158	11.7±1.2^b^	19.7±1.3^b^	22.5±2.0^a^	53.9±4.1^b^	66.4±2.7^b^	33.6±2.7^b^
Ascorbic acid (50 µM)	290	10.4±3.6^b^	11.6±2.6^a^	19.7±3.1^a^	58.4±6.4^b^	73.0±2.9^a,b^	26.96±2.9^a,b^
Glutathione (3.0 mM)	234	14.6±0.5^b^	19.80±0.88^b^	24.7±1.4^a^	40.9±3.9^b^	58.7±2.1^b^	41.3±2.1^b^
Melatonin (10^-5^ M)	188	7.6±1.4^a^	8.3±1.4^a^	12.2±1.7^b^	71.9±4.6^a^	82.1±3.3^a^	17.9±3.3^a^

*Differs significantly within the same column at p<0.05

**Figure-5:**
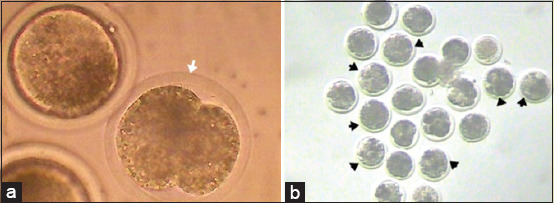
Photomicrograph showing cleaved embryo at 2 cell stage (white arrow) for *in vitro* fertilized buffalo oocytes matured in the presence of 50 μM ascorbic acid (a). Blastocyst stage (black arrows) derived from *in vitro* maturation buffalo oocytes in medium supplemented with 10^-5^ M melatonin (b).

The effect of addition of 50 μM ascorbic acid or 10^-5^ M melatonin to IVM medium of buffalo oocytes on fertilization, cleavage, and blastocyst rates is illustrated in Table-3. Results indicated that the addition of 10^-5^ M melatonin in IVM medium significantly (p<0.05) increased fertilization, cleavage and blastocysts rates when compared with control or 50 μM ascorbic acid groups (Figure-5b). Meanwhile, fertilization, cleavage (Figure-5a), and blastocyst rates were significantly (p<0.05) increased after addition of 50 μM ascorbic acid to IVM medium compared with the control group. Overall, addition of 10^-5^ M melatonin or 50 μM ascorbic acid to IVM medium beneficially improves the developmental competence of buffalo oocytes cultured under OS condition (20% O_2_).

**Table-3:** Effect of addition of 50 μM ascorbic acid and 10^-5^ M melatonin in maturation medium on fertilization, cleavage, and blastocyst rates of buffalo oocytes (Mean%±SEM).

Group	No. of *in vitro* fertilization oocytes	Fertilization rate (%)	Cleavage rate (%)	Blastocyst rate (%)
Control	126	72.2±1.6^b^	66.8±1.7^b^	19.9±1.5^b^
50 µM ascorbic acid	113	77.8±1.7^a,b^	70.8±1.5^a,b^	23.0±1.8^a,b^
10^-5^ M melatonin	134	87.3±1.7^a^	86.6±1.3^a^	31.2±2.0^a^

^a,b^Differ significantly within the same column at P<0.0

## Discussion

*In vitro* production of embryos at oxygen tension close to normal physiological level present *in utero* (5-7% oxygen tension) produced higher cleavage and blastocyst rates [22]. The present data indicated that maturation rate was significantly (p<0.05) higher for buffalo oocytes IVM under 5% O_2_ than that matured under OS condition of 20% oxygen tension. After IVF, cleaved rate was higher (p<0.05) and the percentage of 2-4 cell stage was lower (p<0.05) for buffalo oocytes IVM and IVF under 5% CO_2_ + 5% O_2_ compared to that cultured under OS condition of 5% CO_2_ + 20% O_2_. Furthermore, culture of the presumptive zygotes under 5% CO_2_ + 5% O_2_ increased (p<0.05) the development of buffalo embryos to the blastocyst stage than that cultured under 5% CO_2_ + 20% O_2_. These results indicated that 5% CO_2_ + 5% O_2_ support the development of *in vitro* produced buffalo embryos to the blastocyst stage better than 20% O_2_ conditions. These results are concomitant with those previously reported in cattle [9,23] and pigs [24]. Embryos cultured under OS showed higher ROS accumulation and higher abundance of transcripts related to OS response, which, in turn, decreases cell proliferation and number. In addition, it affects mitochondrial and endoplasmic reticulum functions, resulting in metabolic alterations, DNA fragmentation, and other detrimental effects [9,22]. Low oxygen tension during maturation alters the expression of genes related to bovine oocyte competence and glucose metabolism and significantly improves embryo development, but not blastocyst quality [4,25]. In contrast, other studies reported that oxygen tension did not affect the proportion of one-cell embryos undergoing cleavage or progressing to bovine morula and blastocyst stages [26], sheep and deer [27], or human [28]. This difference could be attributed to species difference or to the conditions under which oocytes or embryos were cultured.

According to our knowledge, this is the first study to address the effect of ascorbic acid, GSH, and melatonin on cumulus cell expansion, nuclear maturation, cleavage, and development of buffalo oocytes *in vitro*. The present investigation revealed that IVM of buffalo oocytes in basic maturation medium supplemented with 10^-5^ M melatonin or 50 μM ascorbic acid significantly (p<0.05) increased cumulus cell expansion and nuclear maturation of buffalo oocytes reaching to the MII stage compared to the control oocytes or those matured in basic medium supplemented with 3.0 mM GSH. Moreover, addition of 10^-5^ M melatonin in IVM medium of buffalo oocytes cultured under 20% O_2_ tension significantly (p<0.05) increased fertilization, cleavage, and blastocyst rates compared with control or 50 μM ascorbic acid groups. The beneficial effects of melatonin on in vitro embryo development have been recorded in mice [29], buffalo [30,31], bovine [32,33], ovine [34], porcine [35], and humans [36]. Melatonin improves cytoplasmic maturation of bovine oocytes by improving the normal distribution of organelles and increasing intracellular GSH, ATP levels, and upregulates genes regulating oocytes maturation [33,37], and mitigates mitochondrial DNA damage [17], and increase total antioxidant capacity in mice oocytes [35]. In contrast, in bovine, melatonin supplementation of IVM media did not improve the cleavage and blastocyst rates [38]. This discrepancy could be attributed to the dose or source or batch of melatonin used for IVM.

Moreover, the present work revealed that addition of 50 μM ascorbic acid into IVM of buffalo oocytes cultured under 20% O_2_ tension did not affect cumulus cell expansion, while it significantly (p<0.05) increased nuclear maturation rate compared with control or GSH groups. It also increased (p<0.05) the blastocyst rate compared with the control one. Addition of Vitamin C to the oocyte maturation medium improves maturation rates of porcine oocytes [39]. The beneficial role for Vitamin C was to protect the spindle structures of MII mouse oocytes and chromosomal alignment against an oxidant (hydrogen peroxide) induced damage [40]. It is suggested that the effect of Vitamin C was associated mainly with its capability to promote ooplasmic maturation during IVM. In contrast, addition of Vitamin C to the oocyte maturation medium has no beneficial effect on the maturation rates of oocytes [41]. This difference could be due to the concentration or culture conditions during IVM. Furthermore, in the present work, the addition of 3.0 mM GSH to IVM of buffalo oocytes adversely affects both cumulus cell expansion and nuclear maturation of IVM buffalo oocytes, and therefore, it is excluded from the study of *in vitro* fertilization and embryo development. This could be in part due to the dose or batch of GSH used and different concentrations of GSH should be tested to identify the proper dose for IVM of buffalo oocytes.

## Conclusion

About 5% O_2_ tension is the optimum gas condition for *in vitro* production of buffalo embryos. However, addition of 10^-5^ M melatonin to IVM medium of buffalo oocytes can alleviate the adverse effect of OS produced by culture of buffalo oocytes under 20% O_2_ tension.

## Authors’ Contributions

ASSA designed the experiments. AME, ASSA, OMKconducted the experiments. ASSA, HHA, AMA performed the statistical analysis. ASSA, OMK, HHA, AME, NEE and AMA drafted and revised the manuscript. All authors have read and approved the final manuscript.

## References

[1] An Q, Peng W, Cheng Y, Lu Z, Zhou C, Zhang Y, Su J (2019). Melatonin supplementation during *in vitro* maturation of oocyte enhances subsequent development of bovine cloned embryos. J. Cell Physiol.

[2] Soto-Heras S, Paramio M.T (2020). Impact of oxidative stress on oocyte competence for *in vitro* embryo production programs. Res. Vet. Sci.

[3] Li W, Goossens K, Van Poucke M, Forier K, Braeckmans K, Van Soom A, Peelman L.J (2016). High oxygen tension increases global methylation in bovine 4-cell embryos and blastocysts but does not affect general retrotransposon expression. Reprod. Fertil. Dev.

[4] Marin D.F.D, Costa N.N, Santana P.P.B, Souza E.B, Ohashi O.M (2019). Importance of lipid metabolism on oocyte maturation and early embryo development:Can we apply what we know to buffalo?. Anim Reprod. Sci.

[5] Goto Y, Noda Y, Mori T, Nakano M (1993). Increased generation of reactive oxygen species in embryos cultured *in vitro*. Free Radic. Biol. Med.

[6] Lim J, Reggio B, Godke R, Hansel W (1999). Development of *in-vitro*-derived bovine embryos cultured in 5% CO_2_ in air or in 5% O_2_, 5% CO_2_ and 90% N_2_. Hum. Reprod.

[7] Elamaran G, Singh K, Singh M, Singla S, Chauhan M, Manik R, Palta P (2012). Oxygen concentration and cysteamine supplementation during *in vitro* production of buffalo (*Bubalus bubalis*) embryos affect mRNA expression of BCL-2, BCL-XL,MCL-1, BAX and BID. Reprod. Dom. Anim.

[8] Correa G, Rumpf R, Mundim T, Franco M, Dode M (2008). Oxygen tension during *in vitro* culture of bovine embryos:Effect in production and expression of genes related to oxidative stress. Anim. Reprod. Sci.

[9] Leite R.F, Annes K, Ispada J, de Lima C.B, Dos Santos É.C, Fontes P.K, Gouveia Nogueira M.F, Milazzotto M.P (2018). Oxidative stress alters the profile of transcription factors related to early development on in vitro produced embryos. Oxid. Med. Cell Longev.

[10] Ashibe S, Miyamoto R, Kato Y, Nagao Y (2019). Detrimental effects of oxidative stress in bovine oocytes during intracytoplasmic sperm injection (ICSI). Theriogenology.

[11] Beheshti R, Mohammadi-Roshandeh A, Giasi Ghalehkandi J, Ghaemmaghami S, Houshangi A (2011). Effect of antioxidant supplements on *in vitro* maturation of bovine oocyte. Adv. Environ. Biol.

[12] Tao Y, Chen H, Tian N.N, Huo D.T, Li G, Zhang Y.H, Liu Y, Fang F.G, Ding J.P, Zhang X.R (2010). Effects of L-ascorbic acid, alpha-tocopherol and co-culture on *in vitro* developmental potential of porcine cumulus cells free oocytes. Reprod. Domest. Anim.

[13] Tian X, Wang F, He C, Zhang L, Tan D.X, Reiter R.J, Xu J, Ji P.Y, Liu G.S (2014). Beneficial effects of melatonin on bovine oocytes maturation:A mechanistic approach. J. Pineal Res.

[14] Lin T, Lee J.E, Jeong J.W, Oqani R.K, Cho E.S, Kim S.B, Jin D.I (2018). Melatonin supplementation during prolonged in vitro maturation improves the quality and development of poor-quality porcine oocytes via anti-oxidative and anti-apoptotic effects. Mol. Reprod. Dev.

[15] Zhang Y, Wang T, Lan M, Zang X.W, Li Y.L, Cui X.S, Kim N.H, Sun S.C (2018). Melatonin protects oocytes from MEHP exposure-induced meiosis defects in porcine. Biol. Reprod.

[16] Lan K.C, Lin Y.C, Chang Y.C, Lin H.J, Tsai Y.R, Kang H.Y (2019). Limited relationships between reactive oxygen species levels in culture media and zygote and embryo development. J. Assist. Reprod. Genet.

[17] Liu Y.J, Ji D.M, Liu Z.B, Wang T.J, Xie F.F, Zhang G.L, Wei Z.L, Zhou P, Cao Y.X (2019). Melatonin maintains mitochondrial membrane potential and decreases excessive intracellular Ca^2+^ levels in immature human oocytes. Life Sci.

[18] Yoshida M, Ishigaki K, Nagai T, Chikyu M, Pursel V.G (1993). Glutathione concentration during maturation and after fertilization in pig oocytes:Relevance to the ability oocytes to form male pronucleus. Biol. Reprod.

[19] de Matos D.G, Gasparrini B, Pasqualini S.R, Thompson J.G (2002). Effect of glutathione synthesis stimulation during *in vitro* maturation of ovine oocytes on embryo development and intracellular peroxide content. Theriogenology.

[20] Sovernigo T.C, Adona P.R, Monzani P.S, Guemra S, Fda B, Lopes F.G, Leal C (2017). Effects of supplementation of medium with different antioxidants during *in vitro* maturation of bovine oocytes on subsequent embryo production. Reprod. Domest. Anim.

[21] Abdoon A.S.S, Gabler C, Holder C, Kandil O.M, Einspanir R (2014). Seasonal variations in developmental competence and relative abundance of gene transcripts in buffalo (*Bubalus bubalis*) oocytes. Theriogenology.

[22] Yoon B.S, Choi S.A, Sim B.W, Kim J.S, Mun S.E, Jeong P.S, Yang H.J, Lee Y, Park Y.H, Song B.S, Kim Y.H, Jeong K.J, Huh J.W, Lee S.R, Kim S.U, Chang KT (2014). Developmental competence of bovine early embryos depends on the coupled response between oxidative and endoplasmic reticulum stress. Biol. Reprod.

[23] Bennemann J, Grothmann H, Wrenzycki C (2018). Reduced oxygen concentration during *in vitro* oocyte maturation alters global DNA methylation in the maternal pronucleus of subsequent zygotes in cattle. Mol. Reprod. Dev.

[24] García-Martínez S, Sánchez Hurtado M.A, Gutiérrez H, Sánchez Margallo F.M, Romar R, Latorre R, Coy P, López Albors O (2018). Mimicking physiological O_2_ tension in the female reproductive tract improves assisted reproduction outcomes in pig. Mol. Hum. Reprod.

[25] Bermejo-Alvarez P, Lonergan P, Rizos D, Gutiérrez-Adan A (2010). Low oxygen tension during IVM improves bovine oocyte competence and enhances anaerobic glycolysis. Reprod. Biomed. Online.

[26] Khurana N.K, Niemann H (2000). Effects of oocyte quality, oxygen tension, embryo density, cumulus cells and energy substrates on cleavage and morula/blastocyst formation of bovine embryos. Theriogenology.

[27] Sánchez-Ajofrín I, Iniesta-Cuerda M, Sánchez-Calabuig M.J, Peris-Frau P, Martín-Maestro A, Ortiz J.A, Fernández-Santos M.D.R, Garde J.J, Gutiérrez-Adán A, Soler A.J (2020). Oxygen tension during *in vitro* oocyte maturation and fertilization affects embryo quality in sheep and deer. Anim. Reprod. Sci.

[28] de los Santos M.J, Gámiz P, Albert C, Galan A, Viloria T, Sonia Pérez S, Romero J.L, Remohï J (2013). Reduced oxygen tension improves embryo quality but not clinical pregnancy rates:a randomized clinical study into ovum donation cycles. Fertil. Steril.

[29] Almohammed N.H.Z, Moghani-Ghoroghi F, Ragerdi-Kashani I, Fathi R, Tahaei L.S, Naji M, Pasbakhsh P (2020). The effect of melatonin on mitochondrial function and autophagy in *in vitro* matured oocytes of aged mice. Cell J.

[30] Nagina G, Asima A, Nemat U, Shamim A (2016). Effect of melatonin on maturation capacity and fertilization of Nili-Ravi buffalo (*Bubalus bubalis*) oocytes. Open Vet. J.

[31] Manjunatha B.M, Devaraj M, Gupta P.S, Ravindra P, Nandi S (2009). Effect of taurine and melatonin in the culture medium on buffalo *in vitro* embryo development. Reprod. Domest. Anim.

[32] Dimitriadis I, Paapanikolau T, Vainas E, Amiridis G.S, Valasi I, Samrtzi F, Rekkas C.A (2005). Effects of Melatonin on *in Vitro* Maturation of Bovine Oocytes. Annual Conference of the European Society for Domestic Animal Reproduction (ESDAR) Murcia, Spain.

[33] Zhao X.M, Wang N, Hao H.S, Li C.Y, Zhao Y.H, Yan C.L, Wang H.Y, Du W.H, Wang D, Liu Y, Pang Y.W, Zhu H.B (2018). Melatonin improves the fertilization capacity and developmental ability of bovine oocytes by regulating cytoplasmic maturation events. J. Pineal Res.

[34] Barros V.R.P, Monte A.P.O, Santos J.M.S, Lins L.B.G, Cavalcante A.Y.P, Gouveia B.B, Müller M.C, Oliveira Junior J.L, Barberino R.S, Donfack N.J, Araújo V.R, Matos M.H.T (2020). Effects of melatonin on the *in vitro* growth of early antral follicles and maturation of ovine oocytes. Domest. Anim. Endocrinol.

[35] Yang L, Wang Q, Cui M, Li Q, Mu S, Zhao Z (2020). Effect of melatonin on the *in vitro* maturation of porcine oocytes, development of parthenogenetically activated embryos, and expression of genes related to the oocyte developmental capability. Animals (Basel).

[36] Nishihara T, Hashimoto S, Ito K, Nakaoka Y, Matsumoto K, Hosoi Y, Morimoto Y (2014). Oral melatonin supplementation improves oocyte and embryo quality in women undergoing *in vitro* fertilization-embryo transfer. Gynecol. Endocrinol.

[37] Pang Y, Zhao S, Sun Y, Jiang X, Hao H, Du W, Zhu H (2018). Protective effects of melatonin on the in vitro developmental competence of bovine oocytes. Anim. Sci. J.

[38] Tsantarliotou M.P, Altanasio L, Rosa A.D, Boccia L, Pellerano G, Gasparrini B (2007). The effect of melatonin on bovine in vitro embryo development. Ital. J. Anim. Sci.

[39] Li Y, Zhang Z.Z, He C.J, Zhu K.F, Xu Z.Y, Ma T, Tao J.L, Liu G.S (2015). Melatonin protects porcine oocyte *in vitro* maturation from heat stress. J. Pineal Res.

[40] Kere M, Siriboon C, Lo N.W, Nyuyen N.T, Chu J.C (2013). Ascorbic acid improves the developmental competence of porcine oocytes after parthenogenetic activation and somatic cell nuclear transplantation. J. Reprod. Dev.

[41] Khazaei M, Aghaz F (2017). Reactive oxygen species generation and use of antioxidants during in vitro maturation of oocytes. Int. J. Fert. Steril.

